# A Highlighted Case for Emphasizing on Clinical Diagnosis for Rare Syndrome in Third World

**Published:** 2017

**Authors:** Fatemeh OWLIA, Mohammad-Hassan AKHAVAN KARBASSI, Roqayeh HAKIMIAN, Mohammad Sadegh ALEMRAJABI

**Affiliations:** 1 Department of Oral Medicine, School of Dentistry, Shahid Sadoughi University of medical sciences, Yazd, Iran; 2 Librarian and Search Literature Officer, School of Dentistry, Shahid Sadoughi University of Medical Sciences, Yazd, Iran; 3 School of Dentistry, Department of Oral Medicine, AJA University of medical sciences, Tehran. Iran

**Keywords:** Palmoplantar keratosis, Early tooth loss, Oculodentodigital dysplasia

## Abstract

Premature tooth loss is a disastrous situation that affects deciduous or permanent teeth era with different causes. It may be attributed to some disorders like Papillon-Lefevre syndrome or coffin-lowry syndrome but because of ambiguous nature, precise diagnosis is not easily possible. Moreover, it has very low incidence and defines by few and limited case series, with vague characters to some extent, confusion in detecting the right diagnosis is a common possibility. Hence, it is expectable to have a wrong diagnosis for this case. In this study, a 5-yr-old boy with chief complaint of early tooth loss despite having blindness in left eye and palmar keratosis is reported, although he had some other manifestation of oculodentodigital dysplasia (ODDD) like ataxia, dysarthria and nail deformity, ignoring other extra and intra oral finding. He was diagnosed as Papillon-Lefevre syndrome already, just because of early tooth loss and palmar keratosis.

## Introduction

Oculodentodigital dysplasia (ODDD) is a hereditary disease that affects the regular development of eyes, face, and limb. ODDD, OMIM #164200 usually presents autosomal dominant disease caused by mutations in the *GJA1 *gene located on chromosome 6 (q21-q23.2). Male and female patients are usually affected in equal numbers in family form while in sporadic forms of ODDD, females seem to be more susceptible (1).

## Case report

**Fig 1:**
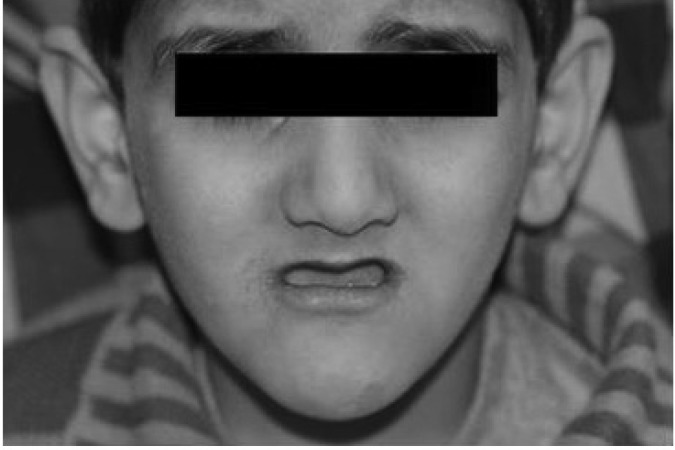
Sever deficiency of maxilla obvious in the image

**Fig 2:**
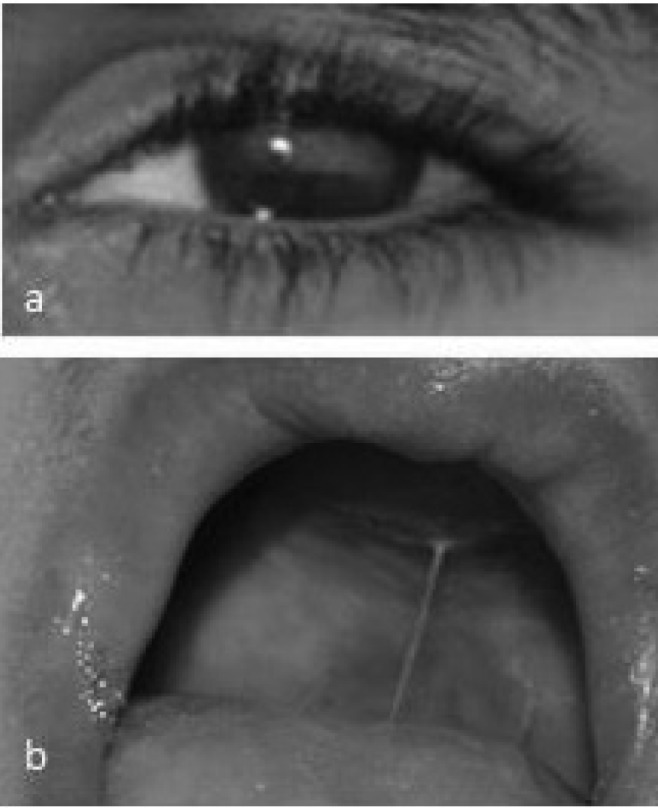
**. **a: A blind eye, b: very small maxillary arch is shown

**Fig 3. a and b:**
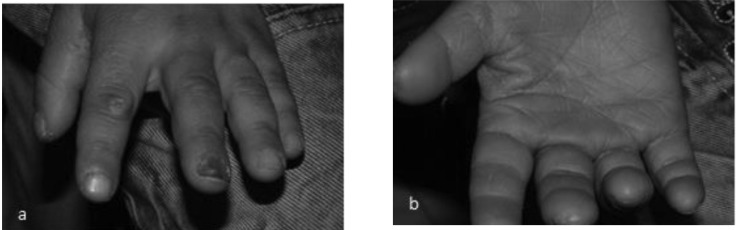
Nail deformity in color and shape and palmar keratosis is shown

A 5-yr-old boy referred to Oral Medicine Department of Yazd (Iran) Dental School, in September 2014, with chief complaint of premature tooth loss since 4 yr ago. He lost his teeth without any trauma. He had problem for speaking and walking. After neurologic consultation, ataxia and dysphonia were confirmed. In his profile small face, severe maxillary deficiency, microphthalmia and blindness in left eye also were detectable (Figure 1). The deformed nails and palmoplantar pit and keratosis were detected. In oral examination, just mandibular incisors were remaining with grade 1 mobility albeit (Figure 2, 3).

A written informed consent was signed by his parents to participate in the study.

## Discussion

Palmoplantar keratosis is the most prominent feature of Papillon-Lefevre syndrome. It should not be considered as a part of this syndrome (just a solitary article reported a Papillon-Lefevre syndrome with eye involvement) (1). His parents were cousins (a traditional common custom); no similar case was seen in his family. Because of close relationship between parents, all of mucocutaneous dyskeratosis with pattern of autosomal recessive could be laid in differential diagnosis. A rational alternative diagnostic option is genodermatoses disease that may lead to blindness is hereditary benign intraepithelial disease (HBID) (2), Because of presence of palmoplantar keratosis this disorder was ruled out. According to ophthalmologist, the etiology of blindness was corneal keratosis.

ODDD is common in Europeans compared to Africans or Asians. They usually were diagnosed in second decade and sporadic form is common in females (sex ratio male/female was close to 6:15) (3). Therefore, this case is very rare for someone, who is an Iranian young male in sporadic form. He was referred to our department with a diagnosis of Papillon-Lefevre syndrome (PLS), because of palmar keratosis and early tooth loss. Considering ocular involvement is not a part of PLS (2), in absence of other affirmative documents for PLS, obviously the diagnosis was questionable.

An article was discussed about ocular involvement of PLS.6 after more research it turned out, palmoplantar keratosis explained in the article, indeed was keratoderma (4). Palmoplantar keratosis is the most prominent property of PLS. Vohwinkel syndrome, Pachyonychia Congenita, and Darier disease share this feature with each other (5). 

Our database lists above syndromes as having palmoplantar keratosis as a sign of disorders. Less frequency of a syndrome could be misdiagnosed easily with more popular one. Unexperienced clinicians may think it was another variant of more prevalent syndromes with different gene expression. Pachyonychia congenital defined by presenting such criteria: some signs like palmoplantar keratosis, special nail deformity, hyperhidrosis and oral leuko- keratosis. Nail deformity appears soon after the birth, therefore, in such case with no sign of related deformity in this age, the probable diagnosis was ruled out.

Darier disease is a skin condition with major presentation of firm, yellow skin blemishes that lead to strong odor. They appear usually on scalp arms, knees and behind the ears. They become worse in humid and hot situations. Other features consist of palmar keratosis in shape of line or pit. In some involved patients, neurologic problem like depression is seen. There is no evidence of early tooth loss in this syndrome; therefore, drier disease was ruled out. The other presumptive diagnose could be Vohwinkel syndrome with classic criteria as follows:

1. Diffuse palmoplantar keratoderma in honeycomb pattern.

2. Constricting bands of digits with auto amputation.

3. Starfish shaped hyper keratotic plaques on the dorsum of hands and feet, elbows and knees.

4. Scarring alopecia and non-progressive sensorineural hearing loss.

Sometimes infants with Vohwinkel syndrome has a collodion membrane that consists of clear sheet on the skin (6). 

The patient characteristic did not match with these criteria. Another possible diagnosis was ectodermal dysplasia because of having no evidence of hair loss or impaired hearing this diagnosis was omitted. In sum, despite its severity and absence of classic criteria of ectodermal dysplasia, oculodentodigital dysplasia is a more reasonable diagnosis. This is one of rare disorders. 

Oculodentodigital dysplasia can affect many parts of the body. From small eye to vision loss, Ocular involvement has a wide range. Some presentations of this disorder in teeth are missing teeth, thin enamel, and premature tooth loss. Less common manifestations of the disorder are thin hair, fingers with abnormal curves and microcephaly. The patient may suffer from ataxia, spastic muscles, and difficult speech. Another sign of ODDD is cardiac abnormality that involved fewer than five percent of patients (1). 

A 5 yr old boy was referred with chief complaint of multiple decayed teeth and tooth abnormality. He was diagnosed without any genetic study while in this case palmar keratosis and early tooth loss were the prominent presentations of patient (3). A 20-day newborn as ODDD, was diagnosed with hereditary glaucoma. Her mother involvement was a good guide to put provisional diagnosis for her but in this case, there was no similar case in his family (7). Indeed, he was a new case. All of the last case reports represent syndactyly in hands with or without toes involvement, but in this case, there was no evidence of it because no reference has acclaimed it as obligatory criteria for diagnosis, this report was a varied presentation of this disorder. Some patients with oculodentodigital dysplasia experienced the condition named “palmoplantar keratoderma”. It means condition that skin of palms and the soles of feet become rough and coarse. Some features of oculodentodigital dysplasia are evident at birth, while others may be apparent with growing up. This condition usually inherited in autosomal dominant and less commonly autosomal recessive. Some cases appear with de novo mutations while there is no history of the disorder in their family tree (8). 

Early diagnosis of rare syndromes like ODDD, sometimes will allow clinician to prevent other probable difficulties. In addition, it will enable better management of associated disease to improve quality of life of the patient.

**In Conclusion, **because of wide spectrum of signs and symptoms, craniofacial syndromes pose a diagnostic challenge for clinician. A systematic approach is necessary for correct diagnosis of syndromes with different manifestations. Patient's features were fulfilled all of criteria for final diagnosis of syndrome, having low economic resource patients, concerning about cost of para clinic tests, it could be postponed to demanded clinical step.
